# Functioning of autobiographical memory specificity and self-defining memories in people with cancer diagnosis

**DOI:** 10.7717/peerj.8126

**Published:** 2019-12-19

**Authors:** Marta Nieto, Beatriz Navarro-Bravo, Beatriz Moreno, Alberto Ocana, Juan Pedro Serrano, Clotilde Boix Gras, Jorge Ricarte, Luz Fernández-Aguilar, Laura Ros, Jose Miguel Latorre

**Affiliations:** 1 Department of Psychology, University of Castilla-La Mancha, Albacete, Spain; 2 Applied Cognitive Psychology Unit, University of Castilla-La Mancha, Albacete, Spain; 3 Unidad de Investigación, Gerencia de Atención Integrada de Albacete, Fundación del Hospital Nacional de Parapléjicos, Albacete, Spain; 4 Asociación Costuras en la Piel en Apoyo a la Unidad de Investigación de Cáncer, ACEPAIN, Albacete, Spain; 5 Translational Research Unit, Albacete University Hospital, and CIBERONC, Albacete, Spain; 6 Centro Regional de Investigaciones Biomédicas, University of Castilla-La Mancha, Albacete, Spain; 7 Centro de salud zona 8, Servicio de Salud de Castilla-La Mancha, Albacete, Spain

**Keywords:** Cancer, Depression, Stress, Autobiographical memory specificity, Self-defining memory, Executive functions, Perceived stress

## Abstract

**Objectives:**

Cognitive and emotional disturbances have been associated with the diagnosis and treatment of cancer. Autobiographical memory is one of the specific cognitive processes affected during this disease. The current study had two main aims: (1) to compare the functioning of autobiographical memory specificity and its related variables (executive functioning, depression and perceived stress) in a group of persons with cancer and a control group; and (2) to analyze whether the experience of cancer evolved into a self-defining memory in the sample of participants diagnosed with this disease.

**Method:**

The study sample comprised 62 participants, 31 in the group with a cancer diagnosis and 31 in the control group. Autobiographical memory specificity, executive functions, depression, stress and self-defining memory were evaluated in the current study.

**Results:**

Depressive symptomatology and reduced executive functioning, but not perceived stress levels, are related and are predictors of autobiographical memory specificity. In addition, the identified characteristics of the self-defining memories were associated with the cancer experience as a threat to physical integrity and an awareness of the meaning of life.

**Conclusion:**

This emerging research line is especially important in view of its possible impacts on patients’ well-being, due to the importance of psychological processes in cancer disease.

## Introduction

Cancer continues to be one of the main causes of mortality in the world (World Health Organization, 2016), which means that studies on this disease are especially important. In Spain, the records of the Spanish Society of Medical Oncology (2019) show that this disease is one of the primary causes of death, with colorectal, prostate, lung, breast, bladder and stomach cancers being the most frequent diagnoses. As well as being considered a life-threatening event, the cancer experience also involves multiple stressors related to the different phases that a patient has to go through, such as diagnosis, treatment and the post-treatment effects, as well as the progression or relapse of the disease (Andrykowski & Kangas, 2010; Parikh et al., 2015; Przezdziecki et al., 2013; Pyter, 2016). Depressive states and mood-related disorders are thus common reactions among patients (Caruso et al., 2017).

Cancer also generates major psychosocial challenges that impact the economic, physical, psychological and social well-being of patients (Jacobsen & Andrykowski, 2015). In most cases, the disease involves the patient having to take on a new personal role with implications for their psychological adjustment, especially when the cancer experience becomes central to their identity (Gökler-Danışman, Yalçınay & Yiğit, 2017; Park, Bharadwaj & Blank, 2011). When people recall their past in association with life-threatening concerns or conflicts such as a disease process, their self-defining memories (SDMs), which are memories that help oneself and significant others to understand who one is as a person (Singer & Moffitt, 1992), tend to be representative of these events. They have an integrative function because they contain lessons about the self or the world beyond the remembered events (McLean & Thorne, 2003). This integrated function may indicate that the individual engages in the construction of a life story and uses the past to inform a sense of identity (Blagov & Singer, 2004).

Among the most significant psychological changes associated with the disease, a large percentage of persons report cognitive disturbances related to chemotherapy and hormone therapy. In this case, the most commonly affected domains are memory, processing speed, and executive functioning (Huehnchen et al., 2019; Li & Caeyenberghs, 2018; Ono et al., 2015). Specific mechanisms underlying therapy-induced cognitive deficits are multifactorial and largely unknown, because a variety of other processes may contribute to them, such as genetic, age-related, emotional, social and behavioral factors, treatments, cancer-related fatigue, presence of comorbidity, hormonal changes or pre-existing cognitive deficits (Vitali et al., 2017; Wefel et al., 2010).

The cognitive process of autobiographical memory (AM) is affected in persons who suffer from cancer (Giffard et al., 2013). AM refers to a knowledge base of personal information that includes specific episodic memories of past events and more conceptual, self-related information (Conway & Pleydell-Pearce, 2000). A characteristic of autobiographical knowledge is its hierarchical organization across different levels of specificity (Conway & Pleydell-Pearce, 2000). At the general level, it can find extended memories, which are general memories associated with events that last more than a day (e.g., “the time I spent in treatment for a medical problem”), and categorical memories, which are general, repeated events grouped together in a category (e.g., “Saturday-night dinners with friends”). At the lowest level of the hierarchy are specific AMs, which are personally significant memories associated with a time and place that lasted a day or less than a day (e.g., “the day my child was born”).

Difficulty in retrieving specific AMs (i.e., a cognitive style consisting of retrieving general memories from the AM, commonly known as overgeneral memory) is one of the most widely studied aspects of AM, due to its association with certain types of psychopathology, such as depressive disorders or post-traumatic stress disorder (Ono, Devilly & Shum, 2016; Williams et al., 2007). AM performance is clearly related to a positive emotional evolution in depression (Williams et al., 2007) and social problem solving (Barry et al., 2018; Williams, 2006) skills. In addition, the mature integration in the self of a negative experience in life is an observed outcome in well adapted post-disease patients (Martínez-Hernández & Ricarte, 2018). Thus, the exploration and the guide for a correct use of these autobiographical resources in patients with cancer could be considered a cognitive resilience factor to be reinforced in this field. Likewise, reduced executive function resources may influence the retrieval of specific memories (Conway & Pleydell-Pearce, 2000; Williams et al., 2007). Empirical research corroborates that the lack of AM specificity appears to be especially associated with deficits in inhibition, working memory, ability to update and maintain information in working memory, and verbal fluency (Sumner, 2012, for a review). In addition to executive functioning, rumination and emotional avoidance have also been posited as processes that, alone or in conjunction, underlie deficits in AM specificity (see CaRFAX model; Williams, 2006). It has been suggested, for example, that emotional avoidance explains overgeneral memory in cancer survivors (Sansom-Daly et al., 2018).

To date, few studies have focused on AM functioning in persons with cancer. The existing studies have found deficits in AM specificity in patients with breast cancer, gastrointestinal cancer, lymphatic cancer, lung cancer, gynecological cancer and head and neck cancer (see Giffard et al., 2013, for a review; Morel et al., 2015). Interestingly, lack of specificity was identified in patients with psychiatric symptoms (Brewin et al., 1998; Kangas, Henry & Bryant, 2005), and comparing healthy controls with cancer patients (Bergouignan et al., 2011; Nilsson-Ihrfelt et al., 2004). In remitted breast cancer patients, autobiographical deficits have been related to a reduced volume of the posterior hippocampus (Bergouignan et al., 2011). Moreover, Morel et al. (2015) analyzed the psychological impact of breast cancer on AM functioning before initiating adjuvant therapy. The level of anxiety affected specificity: the most anxious patients retrieved fewer emotional details than controls, while the least anxious patients performed at a similar level to controls. More recently, Sansom-Daly et al. (2018) examined life narratives and future-thinking processes among adolescent/young adult cancer survivors, concluding that while survivors’ life events were more specific, negative and illness-focused than controls, their future thinking styles were more general. Consequently, these findings highlight that the impact of the illness underlies cancer survivors’ autobiographical thinking processes, that is, they continue to be “cognitively stuck” in their cancer experiences, with reference to both the past and the future.

### The current study

It has been suggested that AM functioning serves to construct identity and maintain a sense of self over time, and allows us to develop, maintain and improve interpersonal relations. It helps in problem solving and directs future behavior (Bluck & Alea, 2011). In addition, AM is related to well-being as it facilitates adaptive behavior and personal development (Waters, 2013), being considered a mechanism of emotional and cognitive protection (Williams et al., 2007). Thus, given that cancer, like other life-threatening diseases, presents physical and psychological challenges (Park, Bharadwaj & Blank, 2011; Przezdziecki et al., 2013) that could affect patients’ AM functioning and their well-being, we propose two aims in the present study: (1) to compare AM functioning and its related variables in a group of persons with a cancer diagnosis and a group of healthy controls; and (2) to analyze whether the cancer experience evolves into a SDM in the sample of patients.

Thus, considering previous studies in which AM specificity deficits were found in persons with a cancer diagnosis (Giffard et al., 2013), we expect the number of specific autobiographical memories retrieved in the people with cancer group to be significantly lower than in the control group. Additionally, difficulty in retrieving specific memories is associated with depressive symptoms and reduced executive functions (Williams et al., 2007). Finally, aware of the psychosocial challenges faced by persons with a cancer diagnosis and considering their possible effects on identity (Gökler-Danışman, Yalçınay & Yiğit, 2017; Jacobsen & Andrykowski, 2015), we hypothesize that the cancer experience will evolve into a highly central and significant autobiographical memory in our sample of diagnosed participants.

## Method

### Participants

The study sample comprised 62 participants, 31 in the group with a cancer diagnosis and 31 in the group without a diagnosis (age range = 37–75 years; *M* = 51.78, SD = 9.28). Table 1 shows the sociodemographic characteristics and the medications taken in both groups. With regard to the latter, 35.8% of the cancer group participants were taking no medication; 29.03% were on anti-depressants and/or tranquilizers; 9.68% were taking painkillers; 9.68%, were taking osteoporosis drugs; and 41.93% were taking cancer medication. A total of 58.06% of controls were taking no medication; 19.35% were on anti-depressants and/or tranquilizers; 3.23% were taking osteoporosis drugs; 3.23% were on anti-cholesterol medication; and 16.13% were following other courses of medication.

**Table 1:** Sociodemographic characteristics of the study sample. The *p* shows significance levels based on Chi-squared test group differences, *t-*test (age) and Mann–Whitney test (number of drugs) group differences; people with cancer group (*n* = 31); non-cancer group (*n* = 31).

Variable	People with cancer group		Non-cancer group		*p*
	*n*	%	*n*	%	
Gender (females)	28	90.3	28	90.3	1
Education level					0.680
No studies completed	1	3.2	0	0	
Primary	6	19.4	8	25.8	
Secondary/vocational training	13	41.9	10	32.3	
University	11	35.5	13	41.9	
Age (*M*, SD)	51.78 (9.36)		51.79 (9.37)		0.996
Number of medications (*M*, SD)	1.39 (1.58)		1 (1.69)		0.150

The participants with a cancer diagnosis were recruited from a cancer research support association (*Asociación Costuras en la Piel en Apoyo a la Unidad de Investigación de Cáncer*, *ACEPAIN*) located in a city of Spain. The inclusion criteria for the study were: (1) being diagnosed with cancer; (2) being sufficiently literate to be able to answer the self-complete questionnaires; and (3) once informed of the study aims, agreeing to participate and signing an informed consent form. The control group comprised 31 participants without a cancer diagnosis, but of similar characteristics to the cancer people group in terms of age, gender and educational level. The controls were recruited from a group of patients at a primary care center belonging to the health service of Castilla-La Mancha (Spain). The inclusion criteria were the same as those for the group of patients, with the exception that they should never have been diagnosed with cancer. An exclusion criterion for both groups was the presence of physical or psychological problems that might prevent their understanding and completing the questionnaires. No participant was excluded for this reason.

### Measures

#### Written Autobiographical Memory Test

The Written Autobiographical Memory Test (AMT) (Raes et al., 2003) was used to assess participants’ capacity to retrieve specific autobiographical memories. The task is based on the presentation of 10 cue words varying in emotional valence, five positive (happiness, friendship, excitement, energy and smile) and five negative cue words (guilty, failure, worry, sadness and illness), for which participants are asked to retrieve a specific memory and write it down in a booklet. Prior to the test, the concept of specific memory was explained (i.e., personal event or situation associated with a specific time and place that lasted less than 24 h) and participants were also given two practice words (car and tree) to ensure their understanding of the instructions. They were given a minute to answer and if no memory was generated after this period, they were instructed to go on to the next word. All the memories were coded following the guidelines proposed by Williams (1992). Memories were coded as specific when they referred to a specific place and moment lasting less than a day (e.g., “when I won a lottery prize”). Events or situations repeated during a certain period of time (e.g., “when I used to go to my parents’ house”) or memories referring to events taking place over a prolonged period of time (e.g., “when I lived in Madrid”) were classified as general memories. Finally, all the responses that could not be classified as specific or general memories were categorized as no memory. The memories were coded by three separate examiners blinded to the objectives of the study and who had not participated in the data collection. To calculate the percentage of inter-rater agreement on the coding of the AMT, two researchers independently evaluated 100 randomly selected memories. They agreed on the categorization into specific or general memories in 93% of the cases, with a Kappa coefficient of 0.858.

#### Contents of autobiographical memory: self-defining memories

A self-defining memory is a highly central and significant personal memory that evokes strong emotion and sensory details at the time of recollection (Singer & Moffitt, 1992; Singer, Rexhaj & Baddeley, 2007). Participants are asked to provide a self-defining memory meeting the following criteria: (1) it is from at least a year ago; (2) it refers to an event from their own lives that they remember very clearly and that continues to be important for them; (3) it is a memory about something important over time, a situation, or a life conflict that helps to explain who they are; (4) it is a memory related to other similar memories on the same subject or field of interest; (5) it may be positive or negative, or both. The main thing is that it generates strong feelings; and (6) it is a memory they have thought about many times and which is familiar to them. After writing a description of the memory, participants are asked to answer 22 questions about the characteristics of the memory on a scale from 1 to 7. Items 1–3 focus on the clarity of the episode recalled in the participant’s memory, with scores ranging from 1 (vague) to 7 (clear). The dichotomous item 4 asks whether the memory has a positive or negative value for the respondent, while item 5 assesses the intensity of this valence (1 to 7). Item 6 refers to whether the individual remembers the event from an observer perspective or actor perspective, or both. Items 7 to 22 evaluate the implications of this remembered event in the individual’s life, scored on a scale from 1 (not at all) to 7 (very much).

#### Test de los Senderos (trail test)

The *Test de los Senderos* (TESEN; Portellano & Martínez, 2014) is based on tests such as the trail making test (Reitan, 1992). It comprises four different trails of increasing difficulty that are administered consecutively to assess the main executive components (e.g., inhibition, working memory and cognitive flexibility). TESEN also includes processing speed, as the trails must be completed as fluently as possible. Trails 1 and 2 are those with less executive demand, being relatively simple tasks related to processing speed. Executive functioning intervenes more directly in Trails 3 and 4. In each of the trails, both accuracy (*P* = (correct-incorrect/correct) × 100) and speed (time in minutes), and a total execution score is provided (*E*_total_ = (correct-incorrect/speed) × 100). Given that the tasks are simple, there is no time limit and the errors are few, the faster the tasks are executed, the better the performance is. The internal consistency of the TESEN are adequate: Speed = 0.93; Accuracy = 0.89; and Execution = 0.88.

#### Digit Span

The Digit Span subtest from the Wechsler Intelligence Scale for Adults was used to assess working memory (Wechsler, 2012). The test comprises three tasks: Digit Span Forward (DSF), Digit Span Backward (DSB) and Digit Span Growing (DSG). The tasks are individually administered. In the DSF task, the examiner reads a sequence of digits of increasing length, which the participant is asked to repeat from memory in the same order. In the DSB task, the examiner reads a sequence of digits and the participant is asked to repeat the digits in the reverse order from the spoken sequence. Finally, in DSG, the examiner reads a sequence of digits that the participant must repeat from memory in ascending order. For the three tasks, the length of the sequence varies between two and nine digits (eight sequences) and two different sequences are presented for each length (16 different sequences). The tasks end when the participant is unable to remember either of the two same-length sequences.

#### Beck Depression Inventory

The Beck Depression Inventory (BDI; Beck, Steer & Brown, 1996) comprises 21 items that identify depressive symptoms. Each item is scored on a four-point Likert-type scale, and the participant is asked to respond with reference to the week prior to completing the questionnaire. Higher scores indicate greater presence of depressive symptoms. Scores range from a minimum of 0 to a maximum of 63. The self-administered version of the BDI has an alpha coefficient of 0.86 (Beck & Steer, 2006).

#### Perceived Stress Scale

The Perceived Stress Scale (PSS; Cohen, Kamarck & Mermelstein, 1983; Spanish version by Remor, 2006) is a self-report instrument that assesses the perceived level of stress during the last month. It consists of 14 items that individuals rate on a five-point scale (0 = never; 1 = almost never; 2 = sometimes; 3 = fairly often; 4 = often). The higher the score, the higher is the level of perceived stress. PSS has shown adequate reliability (α = 0.81).

#### Coping with Stress Questionnaire for Cancer Patients

The Coping with Stress Questionnaire for Cancer Patients (CAEPO; González, 2015) is an instrument that collects information on the behavioral and cognitive coping strategies used by patients in response to a situation of stress caused by cancer diagnosis, therapies and other social and family situations related to the disease. The questionnaire consists of 40 items which individuals rate from 0 to 3 (from *never* to *almost always*) and which are organized in seven scales including approach and avoidance coping strategies. The scale yields an overall score that determines the individual’s coping style. CAEPO has shown adequate psychometric properties (α = 0.81) and adequate concurrent validity.

### Procedure

The procedure for this study was approved by the The Albacete Integrated Care Management Clinical Research Ethics Committee (Record N° 11/2015 of the CEIC). First, we held an informational meeting with the management team of the cancer association, and having received consent, we started to contact members to request their collaboration. The individuals who showed interest in learning more about the research, were given more detailed information about the study. Those who agreed to participate were asked to give their informed consent, to safeguard anonymity, among other reasons. They signed the informed consent forms before beginning the first assessment session. As well as consenting to participate in the study, the participants in the people with cancer group were asked to consent to the oncologist on the research team extracting from their medical records the necessary information on the variables related to their cancer. The controls were recruited at primary healthcare centers, by inviting individuals who met the inclusion criteria to participate in the study. Data collection was conducted in two assessment sessions lasting approximately 60 min. The first was a group session, where, using self-complete questionnaires, the participants reported data on sociodemographic variables, autobiographical memory using the AMT, depression and stress. The second session was individual; health related data was collected, and the SDM and executive function tasks were administered. Both sessions were implemented with an interval of no more than 14 days. The assessments were conducted in the facilities of the cancer association and those of the hospital research unit according to each participant’s preferences. Three trained researchers were responsible for collecting the study data. The interviewers were randomly assigned to participants using a random number generator.

### Data analysis

The Statistical Package for Social Sciences (IBM SPSS 24.0) was used for all analyses. First, descriptive analyses were conducted on both groups using measures of central tendency in the case of metric variables, and proportion description in the case of qualitative variables. Second, inter-group comparisons were performed for the main study variables, using the t-student test in metric variables and Chi-squared test on qualitative variables. Third, bivariate correlational analyses were conducted for the total study sample and for the cancer group. Finally, three linear regression models were applied, incorporating the number of specific memories assessed using the AMT as dependent. In the first model, the independent variable was the participants’ group (with the cancer free group as reference). In the second model, using the backward stepwise approach, we incorporated the participants’ group and depressive symptomatology. In the third and last model, as independent variables, as well as the study group, we incorporated depressive symptomatology and executive functioning (DSB, DSG and overall performance on the TESEN).

## Results

### Descriptive results

Of the 31 patients in the cancer group, 25 had been diagnosed with only one tumor and the other six with two. Table 2 shows the characteristics of this group. Coping strategies in the people with cancer group were assessed using the CAEPO scale: 3.6% used mostly negative strategies (positive and negative strategies, with a greater presence of negative ones); 3.6% were non-defined; 46.4% used mostly positive strategies (positive and negative strategies, but with a greater presence of positive ones); and 46.4% used positive strategies (exclusively positive strategies or a clear dominance of positive ones).

**Table 2:** Characteristics of the group with cancer diagnosis.

Variable	*n*	%
Number and location of tumors		
Single	25	80.65
Breast	19	61.29
Colon	3	9.68
Prostate	1	3.23
Thyroid	1	3.23
Lymphatic	1	3.23
Two	6	19.35
Two breast	3	9.68
Breast and lymph	1	3.23
Breast and lung	1	3.23
Uterus and colon	1	3.23
Participants with metastatic tumors	7	22.58
Previous chemotherapy	25	80.65
Chemotherapy currently	1	3.23
Previous radiotherapy	22	70.97
Radiotherapy currently	0	0

Variable	Mean	SD	Range
Time since last cancer diagnosis	3.61	2.86	0–9.75
Time since the first cancer diagnosis	5.51	5.40	0.39–28.51
Time without chemotherapy	4.63	4.07	0–17.73
Time without radiotherapy	3.49	2.88	0.16–10.83

**Note:**

*n* = 31.

### Inter-group comparisons

We found *s*tatistically significant differences between the two groups in AM scores, assessed using the AMT and SDM. Table 3 shows the performance on the AMT for both patients and controls. Regarding AM specificity, the cancer diagnosis group retrieved fewer specific memories (*M* = 5.32, SD = 2.56) compared to the controls (*M* = 6.77, SD = 2.67; *t* = −2.19, *p* = 0.033). We found no statistically significant differences between groups in the valence of the memories. The themes of the memories were also analyzed.

**Table 3:** Autobiographical memories assessed using the autobiographical memory test.

Variable	People with cancer group Mean (SD)	Non-cancer group Mean (SD)	*t*	*p*	Cohen’s *d*
Specific memory	5.32 (2.56)	6.77 (2.66)	−2.19	0.033	−0.55
General memory	3.23 (2.12)	2.29 (2.05)	1.76	0.083	0.45
No memory	1.45 (1.23)	0.94 (1.09)	1.74	0.086	0.44
Valence of the specific memory					
Positive memory	2.55 (1.48)	3.29 (1.49)	−1.97	0.054	−0.50
Negative memory	2.77 (1.50)	3.35 (1.38)	−1.59	0.118	−0.40
Semantic content of the memory					
Related to the participant’s cancer	2.23 (1.50)	0.10 (0.40)	7.65	<0.001	1.94
Related to another person’s cancer	0.35 (0.61)	0.52 (0.68)	−0.987	0.328	−0.26
Non cancer-related	6.19 (1.68)	8.52 (1.26)	−6.15	<0.001	−1.57

**Note:**

People with cancer group (*n* = 31); non-cancer group (*n* = 31).

The analyses of the SDMs showed statistically significant differences between the groups in 7 of the 23 items of the SDM scale, with the highest scores found in the people with cancer group (see Table 4). Regarding the semantic content of the memory reported on this scale, 45.2% of the memories were unrelated to cancer, 38.7% of memories were related to the participants’ own cancer experience, and 16.1% were related to the cancer experience of others. In the control group, 93.5% of memories had no association with cancer, and 6.5% were related to the cancer experience of another person; Fisher’s exact statistic = 20.14; *p* < 0.001.

**Table 4:** Differences in the results on the self-defining memory scale.

Variable	People with cancer group Mean (SD)	No cancer group Mean (SD)	*t*	*p*	Cohen’s *d*
1. Age when the event in the memory occurred	38.48 (14.79)	31.00 (13.15)	2.12	0.039	0.53
3. Level of detail of the memory	6.65 (0.71)	6.03 (1.40)	2.17	0.035	0.56
4. Clarity in the order of events	6.68 (0.75)	6.13 (1.23)	2.12	0.039	0.54
9. Memory of how they felt at that time	6.94 (0.25)	6.19 (1.68)	2.43	0.021	0.62
14. To what point was their physical integrity threatened	4.10 (2.69)	2.00 (1.73)	3.65	0.001	0.93
20. Learning about the meaning of life	6.84 (0.45)	6.26 (0.93)	3.12	0.003	0.79
22. Extent to which they notice they feel affected	5.81 (1.80)	4.68 (2.06)	2.30	0.025	0.58

**Note:**

People with cancer group (*n* = 31); non-cancer group (*n* = 31).

To control for possible confounding variables, we assessed values such as levels of depression, stress and aspects related to executive functioning. Table 5 shows the differences between groups. The results of the BDI, DSB, DSG and TESEN revealed statistically significant differences between the two groups in the levels of depressive symptomatology. The scores on the TESEN showed no statistically significant differences in the number of correct responses, but the differences in execution times were found to be statistically significant.

**Table 5:** Inter-group differences in depressive symptomatology and executive functioning.

Variable	People with cancer group Mean	Non-cancer group Mean	*t*	*p*	Cohen’s *d*
BDI	14.87 (11.53)	8.52 (7.11)	2.61	0.012	0.66
PSS	25.35 (9.85)	22.32 (10.13)	1.20	0.237	0.30
Working memory					
Digit span forward	5.61 (1.12)	6.10 (1.11)	−1.72	0.092	−0.44
Digit span backward	3.94 (1.18)	4.81 (1.30)	−2.76	0.008	−0.70
Digit span growing	4.84 (1.46)	6.06 (1.34)	−3.44	0.001	−0.87
TESEN					
Speed trail 1 (minutes)	2:00 (0:40)	1:38 (0:31)	2.61	0.012	0.61
Execution trail 1	0.22 (0.07)	0.26 (0.07)	−2.51	0.015	−0.57
Speed trail 2 (minutes)	2:10 (0:47)	1:45 (0:26)	2.61	0.012	0.66
Execution trail 2	0.20 (0.07)	0.23 (0.06)	−1.77	0.082	−0.46
Speed trail 3 (minutes)	2:20 (0:46)	1:50 (0:29)	3.10	0.003	0.78
Execution trail 3	0.14 (0.05)	0.18 (0.05)	−2.54	0.014	−0.80
Speed trail 4 (minutes)	3:15 (1:22)	2:36 (0:47)	2.30	0.026	0.58
Execution trail 4	0.11 (0.05)	0.12 (0.04)	−1.42	0.162	−0.22
Execution total	0.16 (0.05)	0.19 (0.04)	−2.16	0.035	−0.66

**Notes:**

People with cancer group (*n* = 31); non-cancer group (*n* = 31).

BDI, Beck depression inventory; PSS, Perceived stress scale; TESEN, Trail test.

### Bivariate correlations

Table 6 shows the correlations between the number of specific memories retrieved on the AMT, levels of depression and stress, and executive function performance (digit test and trail test). The number of specific memories was negatively associated with levels of depression and positively associated with executive functioning. In addition, we calculated the correlation in the cancer group between the number of specific memories and age, time since the first cancer diagnosis, time since the most recent cancer diagnosis and time since the completion of chemotherapy and radiotherapy treatments. We found no statistically significant relationships between these variables. Finally, we calculated the correlations between the scores on the CAEPO scale (the higher the score, the more positive the coping style) and memory specificity and valence. The correlation between positive coping style and the number of specific memories was 0.470 (*p* = 0.012) and between positive coping style and the number of positive memories, it was 0.425 (*p* = 0.024).

**Table 6:** Pearson correlation coefficient across the main study variables (*n* = 62).

	1	2	3	4	5	6	7	8	9	10	11
1. Specific memories	–										
2. Depression	−0.345**	–									
3. Perceived stress	−0.194	0.751**	–								
4. Digits forward	0.374**	−0.285*	−0.343**	–							
5. Digits backward	0.441**	−0.314*	−0.346**	0.470**	–						
6. Digits rising	0.306*	−0.196	−0.175	0.363**	0.425**	–					
7. Execution TESEN 1	0.165	−0.229	−0.071	0.263*	0.351**	0.519**	–				
8. Execution TESEN 2	0.293*	−0.382**	−0.220	0.345**	0.415**	0.530**	0.691**	–			
9. Execution TESEN 3	0.292*	−0.325**	−0.150	0.249	0.225	0.534**	0.724**	0.715**	–		
10. Execution TESEN 4	0.222	−0.305*	−0.142	0.221	0.291*	0.553**	0.677**	0.676**	0.670**	–	
11. Execution TESEN total	0.263*	−0.354**	−0.161	0.277*	0.355**	0.613**	0.878**	0.846**	0.875**	0.888**	–

**Notes:**

TESEN, Trail Test.

* *p* < 0.05.

** *p* < 0.01.

### Linear regression analysis

Linear regression was used to analyze the association between belonging to the cancer or control group and memory specificity as assessed on the AMT. A second model, using the backward stepwise approach, was conducted incorporating both group and depressive symptomatology. Finally, a model was run in which, as well as group and depressive symptomatology, we included executive functioning (DSB, DSG and total performance on TESEN). The association between group and memory specificity disappeared when possible confounding variables were added to the model (see Table 7).

**Table 7:** Linear regression models.

	*B*	SE	Beta	*t*	*p*	Adjusted *R*^2^
Model 1						
Constant	8,226	1,05	–	7,833	<0.001	0.058
Group (reference = non-cancer group)	−1,452	0,664	−0,272	−2,186	0.033	
Model 2						
Constant	7.132	0.500	–	14.265	0.000	0.104
BDI	−0.093	0.033	−0.345	−2.845	0.006	
Model 3						
Constant	3.37	1.28	–	2.64	0.011	0.236
BDI	−0.068	0.032	−0.250	−2.11	0.039	
DB	0.785	0.245	0.378	3.20	0.002	

**Notes:**

Only the significant variables are included in the models; dependent variable = specific memories.

BDI, Beck depression Inventory; DB, digits backward.

## Discussion

The present study focuses on two main aims: (1) to compare the functioning of AM specificity and related variables in a group of persons with cancer and a control group; and (2) to analyze whether the cancer experience evolved into a SDM in the sample of participants diagnosed with cancer.

Concerning the first aim, we found that groups differed in the capacity to retrieve specific autobiographical memories. The findings coincide with those of previous studies identifying similar deficits in individuals with cancer (Giffard et al., 2013, for a review; Morel et al., 2015). Fear of recurrence leads cancer survivors to develop autobiographical thinking that typically contains constant references to the disease (Beith et al., 2017). The findings of Sansom-Daly et al. (2018) show that, relative to controls, adolescent/young adult cancer survivors’ memories were more specific and also more negative, and illness-focused, although their ability to imagine their future lives was more general. In this sense, the devastating nature of cancer may have an unavoidable negative impact on psychological outcomes, especially if the illness is present in adolescence or early adulthood, both crucial stages in the development of identity. Similarly, our results showed that the participants with cancer retrieved more memories with semantic content related to the disease (their own cancer or that of other individuals) than the controls. Although the mean number of negative memories was slightly higher than that of positive ones, no statistically significant differences in valence were found between the groups. As for memory specificity, our findings were inconsistent with those of Sansom-Daly et al., as our participants with a cancer diagnosis showed significantly lower AM specificity compared to the controls. As well as the age at the time of illness, another potential explanation of this discrepancy lies in the methodology. While Sansom-Daly et al., used an interview based on life narratives, that is, a process of direct retrieval (spontaneous memories) in which participants are not explicitly asked to recall specific memories, the AMT used in the present study is a generative retrieval task based on eliciting specific memoires using cue words, the procedure of which demands greater effort and a greater quantity of cognitive resources compared to spontaneous recall (Conway & Pleydell-Pearce, 2000). In addition, as suggested by Morel et al. (2015), these findings could be interpreted as a tendency toward avoidance in patients so as to reduce the emotional impact of retrieval of deeply negative experiences, resulting in a more adaptive coping approach during their illness. In the same line, Debeer et al. (2012) and Hermans et al. (2008) posited that reduced specificity might be a defensive avoidance strategy used on certain occasions by individuals without a psychopathology. Drawing on these perspectives, our results also show that positive coping strategies dominated in the people with cancer group, an aspect underlying individuals’ adaptation to stressful situations. This process, however, should be analyzed in greater depth in future research, given that the present study does not include a between-groups comparison of coping strategies. Information on such strategies was only recorded descriptively in the cancer group, where most of the participants exhibited positive coping strategies.

The main factors that seem to affect specificity are emotional distress such as depression and impaired cognitive functioning (Williams et al., 2007). Thus, the higher levels of depressive symptomatology and reduced executive functioning associated with and predicting specificity in the sample of cancer patients coincide, on one hand, with the findings of studies that report the disease induces drastic changes, interferences in life purposes (Abrams, Hazen & Penson, 2007) and multiple losses underlying depression or grief reaction (Gökler-Danışman, Yalçınay & Yiğit, 2017).

Focusing on depression, Brewin et al. (1998) found that severely depressed cancer patients retrieved fewer specific memories than non-depressed cancer patients. Moreover, in the group of depressive patients, we found intrusive memories related to cancer experience and avoidance associated with such events, which suggests the avoidance of repetitive intrusive memories could be associated with problems in AM functioning, a characteristic feature of depressive thinking (Kuyken & Brewin, 1995). Nevertheless, as previously mentioned, deficits in AM have also been detected in individuals without psychiatric pathology. Bergouignan et al. (2011) reported an intimate relationship between a reduced volume of the posterior hippocampus and reduced autobiographical retrieval in remitted breast cancer patients without psychopathology. Interestingly, the patients in their study showed a positive bias, as did the healthy controls. In this regard, it has been suggested that lack of positive bias may be more specific to stress-related pathologies. In other words, these findings suggest that changes or deficits in memory in individuals with an illness such as cancer may present different characteristics to those found in stress-related disorders.

On the other, it has been suggested that chemotherapy and hormone therapy produce cognitive deficits (Dietrich & Kaiser, 2016; Wu & Amidi, 2017). One of the cognitive domains most referenced in this field is that of the executive functions (Vitali et al., 2017; Yao, Bernstein & Rich, 2017). Shifting and working memory are the processes most affected, while inhibition appears to be relatively unaffected (Yao, Bernstein & Rich, 2017). Studies have also shown that treatments are associated with adverse emotional processes and are considered significant predictors of subsequent cognitive functioning (Van Londen et al., 2014; Menning et al., 2015). Thus, the correlational findings on depressive symptomatology and executive functioning seem to point in the same direction in the current study.

Research has reported difficulties in retrieving specific memories among individuals with acute stress disorder and post-traumatic stress disorder (Ono, Devilly & Shum, 2016; Williams et al., 2007). In the context of cancer, it has been suggested that the trauma event itself or the side effects of the treatments would explain such specificity deficits (Giffard et al., 2013). With regard to stress levels, we found no relation between perceived stress and specificity. This suggests that our findings should be taken with caution, given that no stress-related disorders were recorded, and stress levels were not collected during the acute phase of cancer, including diagnosis and treatment. Furthermore, an aspect of interest that could be addressed in future works is the relation between perceived stress levels and depressive symptomatology. For example, research on individuals with human immunodeficiency virus found that memory specificity moderated the impact of perceived stress, such that perceived stress was more strongly associated with follow-up depressive symptoms among those with greater memory specificity (Yanes et al., 2012).

As regards our second aim, focusing on the centrality of the cancer experience, our findings showed, as expected, that this experience evolved into a SDM in practically half the cancer-surviving patients. The SDMs provide an insight into the memory content most closely related to self-identity (Ricarte et al., 2017). Our findings coincide with previous studies demonstrating that cancer survivorship is linked to a process of identity reconstruction, which evolves into an essential element of identity centrality in these persons (Park, Bharadwaj & Blank, 2011). Similar findings have been reported for patients with a diagnosis of mental disorder (Berna et al., 2011) or with addictive diseases (Martínez-Hernández & Ricarte, 2018), suggesting that identity reconstruction after a serious illness might be a transdiagnostic factor for which patients and their relatives should be informed and supported. In the people with cancer group, these identifying characteristics of the SDMs primarily took the form of a threat to physical integrity and awareness of the meaning of life. Identity reconstruction in situations of chronic illness corresponds to an emotion regulation function normally explained by the presence of redemption sequences in which an initially negative experience is valued as an enriching, positive experience (McAdams et al., 2001; Wood & Conway, 2006). The substantial presence of meaningful learning about life in our participants’ SDMs, in comparison with the control group, suggests not only the adoption of redemption sequences but also the presence of meaning making. Consistent with this, a recent qualitative study among patients interviewed 12 months after diagnosis of oral-digestive cancer, 43% tried to understand why they got cancer and 53% declared that cancer changed their view of life, suggesting that cancer survivors make meanings in the areas of existential, social, and personal domains with both positive and negative content (Moye et al., 2018). Importantly, and according to our results, clinicians in this field should explore and specifically help to regulate the feelings and cognitions generated from the threat to physical integrity and the meaning of life to ensure an optimal emotional adjustment through the cancer trajectory in their patients.

### Limitations

This work has several limitations, the first of which are the small sample size and the cross-sectional design. Longitudinal studies with a larger number of participants would be needed to analyze in more detail the intra-subject evolution of each of the study variables. Second, regarding the mechanisms that explain specificity deficits, the current study is exclusively focused on executive functions, and thus, we suggest that future works should include other processes, such as rumination and emotional avoidance, thus enriching our contributions on AM in cancer patients. Third, this work is intended as a preliminary approach to AM functioning in persons with cancer; it would be advisable to homogenize study samples according to age, time since diagnosis, the type and characteristics of their cancers, stages and treatments. Consequently, in order to draw detailed conclusions, these questions should be the subject of more in-depth analysis in future studies. The final limitation is related to the external validity of our results; the participants were a convenience sample, recruited from a single association, so are arguably not representative of the Spanish cancer population. Thus, future lines of research should focus on the replication of our findings in samples with different origins.

## Conclusion

To conclude, this study shows that depressive symptomatology and reduced executive functioning are related and are predictors of specificity deficits in the sample of people with cancer. Moreover, the centrality of the disease is represented by SDMs characterized principally by an adaptive response involving a perceived threat to physical integrity and awareness of what life means or meaning making. Although the previous literature has reported AM specificity deficits in patients with psychiatric diagnosis, a novel finding of the present study is the corroboration of the existence of such deficits in populations without psychopathology, but with an accumulation of life-threatening events. Likewise, there is scant literature on AM functioning in cancer experience; our patient group is larger than that in most of the previous studies and our study is the first to address the issue of SDM together with AM. Thus, the study of the association between AM retrieval in persons with cancer and its possible implications is a field which calls for further research, due to the importance of psychological processes in the disease and the potential consequences for the well-being of those affected.

## Supplemental Information

10.7717/peerj.8126/supp-1 Supplemental Information 1 Database with all the variables used in the study. Open with LibreOffice, R or SPSS.

## Additional Information and Declarations

### Data Availability

The following information was supplied regarding data availability: The data is available in the Supplemental File.
